# Sequential Solidification of Metal Powder by a Scanning Microwave Applicator

**DOI:** 10.3390/ma16031136

**Published:** 2023-01-28

**Authors:** Yoav Shoshani, Tal Weinstein, Zahava Barkay, Eli Jerby

**Affiliations:** 1 Faculty of Engineering, Tel Aviv University, Ramat Aviv 6997801, Israel; 2 Center for Nanoscience and Nanotechnology, Tel Aviv University, Ramat Aviv 6997801, Israel

**Keywords:** microwave processing, additive manufacturing, 3D printing, metal powders, localized microwave-heating, thermal runaway, hotspots

## Abstract

This study examines the fundamental feasibility of sequential metal-powder solidification by localized microwave-heating (LMH) provided by a scanning, all-solid-state microwave applicator. This continuous process is considered for the additive manufacturing (AM) and 3D printing (3DP) applications of metal parts. In previous studies, we employed LMH for the incremental solidification of small batches of metal powder in a stepwise vertical manner. Here, we study a continuous lateral LMH process, layer by layer, in a fashion similar to laser scanning in powder beds, as performed in common laser-based AM systems. LMH solidification at scanning rates of ~1 mm^3^/s is obtained in bronze powder using ~0.25-kW microwave power. The effect is studied here by LMH scanning in one lateral dimension (~20-mm long) in layers, each of ~1–4 mm thickness and ~2–4 mm width (mechanically confined). Imperfect solid bars of ~20×4×5 mm^3^ are obtained with rough surfaces. Their joining in an L shape is also demonstrated. The experimental solidified products are tested, and their hardness and density properties are found to be comparable to laser-based AM products. The capabilities and limitations of the LMH scanning concept for metal-powder solidification are evaluated. The potential feasibility of a solid-state LMH–AM technology is discussed.

## 1. Introduction

Additive manufacturing (AM) and 3D printing (3DP) are essential technologies in a variety of production processes [1,2] aiming to cope with the increasing demands for complex models and custom-made products that lie beyond the capabilities of conventional manufacturing methods. AM of metal parts is commonly performed by powerful lasers or electron beams [3]. In a typical AM process, the product is incrementally built, layer by layer, utilizing a powder bed or a thin wire as the material feedstock. Selective laser sintering (SLS) [4] is an AM technique using a scanning high-power laser as the heat source that locally fuses and binds the metal powder, layer by layer, in order to form the solid 3D object. Electron beam melting (EBM) is another technology for metal AM in which a focused electron beam in a vacuum consolidates the powder in a scanning mode [5], similar to SLM.

Inert gases, such as nitrogen or argon, are often used to prevent oxidation during AM processes [4] and to improve the AM product quality [6]. Both SLM and EBM technologies may employ means for the powder preheating, which expedites the process initiation stage (e.g., a > 1500 °C preheating in ceramic-powder SLM [7]). Another relevant technique is metal injection molding (MIM) [8], in which a fine metal powder is mixed with a plastic binder to form a green part. A thermal process is then applied to remove the binder and to sinter the final metal product. 

Microwaves have been widely used in various industries for decades, e.g., for purposes of heating and material processing [9,10]. Volumetric microwave heating is usually applied to objects in sizes comparable to the microwave half-wavelength (λ/2 ~6 cm at 2.45 GHz) or larger [9,11]. Unlike conventional (conductive) heating processes, the microwave energy is converted to heat inside the irradiated material (due to dielectric, ohmic, or magnetic loss mechanisms). Microwaves have been widely studied as a means for volumetric sintering of ceramics [11,12,13]. Though bulky metals are considered insusceptible to microwaves, it was discovered [14] that metal powders can be sintered by microwaves in molds.

The effect of microwave heating of metal powders is attributed to the magnetic field interaction with the powder particles [15], which induces eddy currents and hence causes Joule heating in the particles’ skin depth (~2 μm in iron, for instance, at 2.45 GHz [16]). This effect is significant for particles in diameters smaller than their skin depth [17], which are isolated by the slight oxidation of their surfaces. In these conditions, microwaves may heat a metal powder up to its melting point, $T_{m}$. In addition to metal-powder sintering and volumetric solidification in molds [14,17], microwaves have also been explored for the joining of metal parts (using powder fillers or other susceptors in between) [18,19] and for various other processes of casting and production of metal objects [20]. 

Microwave sintering processes have been further studied for metal and metal oxide powders, e.g., tungsten [21], copper [22], ferrite powders [23], transparent alumina [24], metallic glass [25], and high-entropy alloys [26]. Additives, such as susceptors and polymeric binders [27], were found useful for enabling microwave absorption in various cases. Volumetric microwave processes are, therefore, currently applicable for a wide range of materials in a powder form, including metals [17]. However, although microwave sintering in molds saves time and energy (and may result in better products compared to conventional techniques), fabrication in molds is more suitable for the mass manufacturing of large series rather than small quantities of custom-made products. The latter requires other, more versatile processes, such as those offered by advanced 3DP and AM technologies [28]. 

The concept of intentional localized microwave-heating (LMH) in sub-wavelength dimensions [29] has also been proposed and investigated for AM purposes [30,31,32,33]. The LMH effect, utilized before in microwave drilling [34] and basalt melting [35] processes, is enabled by a thermal runaway instability due to the microwave–matter interaction. The LMH effect is characterized by a rapid heating within a confined zone (of a ~2–3 mm diameter), which evolves as a molten hotspot. The main condition for LMH is dictated by the temperature-dependent properties of the irradiated material. The LMH effect has also been utilized for plasma ejection from various materials [36,37] and for metal cutting [38]. Synergic combinations of LMH and direct current (DC) result in intensified thermal runaway effects, as demonstrated in [37,38].

The LMH–AM approach was studied by melting a small batch of metal powder, solidifying it while cooling it down, and consolidating it with the previous batch, in an incremental manner [30,31,32]. In these first studies, a ~1-kW magnetron was employed in a waveguide structure. The powder batch was confined either by a mechanical support [30] or by a contactless external magnetic field (for ferromagnetic metal powders) [32]. Other microwave-assisted AM concepts, have been developed in a more volumetric-heating approach for pre- and post-processing of 3D-printed products, including ceramics [39,40], concrete [41], and alloys [42,43]. 

As solid-state generators are becoming more affordable, mainly in the <1-kW range [44], the trend of transition from magnetron-based systems to solid-state generators for microwave heating is driven by the solid-state advantages of controllability, reliability, compactness, lifespan, and overall cost. Due to the significant energy concentration, LMH effects can be implemented by a relatively low power, in the order of ~0.1 kW. This feature has led to the development of various solid-state LMH applicators [45]. Consequently, the LMH paradigm is also extended to compact microwave heaters and to new applications, such as concrete cutting by solid-state LMH [46], ignition of thermite reactions for material processing and combustion [47] (also in oxygen-free environments such as underwater [48] or in space), and to material identification by breakdown spectroscopy (MIBS) [49]. The transition to compact solid-state applicators reduces the size, weight, operating voltage, and energy consumption of the LMH device. This transition also improves the spectral characteristics, tunability, and controllability of the LMH process. 

The all-solid-state LMH–AM process [33] is made possible by the shielding nitrogen atmosphere applied. The inert gas prevents parasitic plasma effects and saves their ineffective power consumption, and this hence improves the overall power efficiency of the LMH–AM process and its efficacy. The LMH–AM feasibility was demonstrated by incremental constructions of simple elements, such as metal pillars, in the aspects of solidification and incremental consolidation of ~10-mm^3^ metal-powder batches, one on top of the other (i.e., 1D stepwise). On this basis, LMH–AM processing of more complicated structures in various orientations is expected to be producible as well. In the present study, we examine the feasibility of metal-powder solidification by the continuous scanning of the LMH applicator (which mimics to some extent the laser scan in SLS processes). The capabilities and limitations of the continuous LMH–AM process are evaluated in the concluding section.

## 2. Methods and Materials

**Figure 1:**
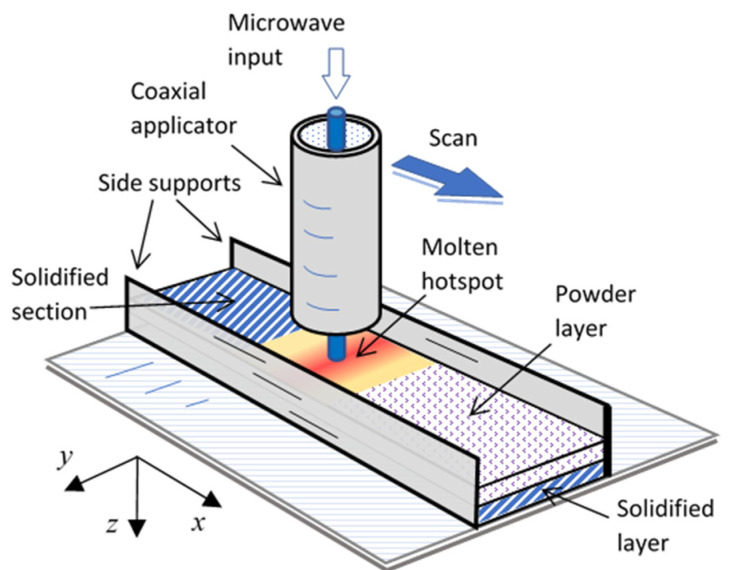
A conceptual scheme of the experimental 1D scanning-LMH–AM process. The powder is applied in layers; each is scanned and solidified by the LMH applicator and bonded to the underlying previously solidified layers.

**Figure 2:**
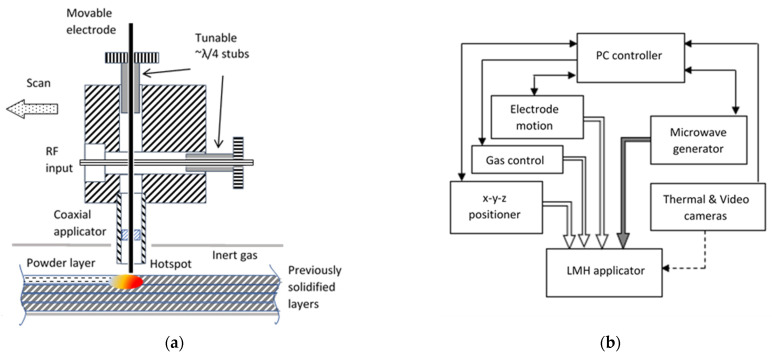
The scanning LMH–AM experimental setup: (**a**) A cross-section of the LMH applicator, including the integrated two-stub tuner and electrode feeder. (**b**) A block diagram of the peripheral equipment and the diagnostics employed in this experiment.

The feasibility of continuous metal-powder solidification by a scanning LMH applicator was investigated using the experimental 1D powder-bed scheme illustrated in Figure 1. The LMH–AM applicator consisted of an open-end coaxial radiator, with an electrode made of a ^Ø^1.6-mm tungsten rod, similar to the microwave drill [34,46]. The electrode’s tip intensified the LMH effect by increasing the local electric field when brought to proximity with the powder layers. The open-end applicator was fed by a cross transition, as shown in Figure 2, which functioned as both a dual-stub tuner and an electrode feeder. The two movable shorts were adjusted to reach an optimal impedance matching with the molten hotspot. They also enabled the inner electrode motion, independent of the outer coaxial tube, via a hole in the center horizontal pin. The electrode feeder provided additional means for the fine-tuning of the LMH impedance matching and for the electrode’s feeding, maintenance, or replacement, as needed.

The applicator was fed by a 2.45-GHz solid-state microwave generator (Pink-RF, MPG500S) via a flexible coaxial cable (N-type, H.S. S07262BD). Its controllable output power was limited in these experiments to 0.3 kW, as this has been commercially available by portable LDMOS and GaN amplifier pallets. The ±50-MHz tunable frequency range of the generator was used here to automatically optimize the impedance matching, by real-time monitoring of the incident and reflected power levels in the adaptive frequency-tuning feedback loop. 

The bronze-based metal powder used in this experiment (DirectMetal-20 EOS, GmbH [50]) was originally designed for laser sintering. The powder was first layered along the 1D bed, as shown in Figure 1. The side supports were made of either plaster, glass–ceramic (MACOR^TM^), or alumina bars (note that these can be replaced by a contactless magnetic confinement for ferromagnetic powders [32]). The LMH–AM interaction was shielded by an inert-gas environment, which prevented the parasitic plasma ejection and hence reduced the total microwave-power consumption (to a level enabling the transition from magnetrons [30] to solid-state generators [33]). No preheating of the powder or its substrate was applied in these experiments.

In order to enable the *x-y-z* motion control (note the coordinates’ definition in Figure 1), the microwave-drill applicator was mounted on a three-axes positioner (a modified CNC machine). The extruder motor of the printer was also used, as the fourth axis, in order to control the electrode motion with respect to the outer tube. The multiaxial motorized positioner allowed for the preprogramming of the applicator’s motion and its scanning sequences, in a variety of shapes and speeds. The process can also be programmed for optimized power transfer and energy saving.

The diagnostic means in this experiment also included a video recorder, a thermal imaging camera (FLIR-SC300, in the range 200–1200 °C), and the internal real-time indications provided by the Pink-RF generator (including the reflection-coefficient, power, frequency, and inner-temperature monitors). The entire solidification process and its diagnostics were controlled and recorded by a LabView program. 

The raw products obtained in the LMH–AM experiment were analyzed ex-situ by a scanning electron microscope, ESEM, FEI Quanta 200FEG (after polishing and etching by Nital solution for 20 s, to expose the microstructure). The chemical element composition was analyzed at an electron beam acceleration voltage of 20 kV using X-ray energy dispersive spectroscopy (EDS) with a silicon drift detector (SDD) (Oxford). The hardness of the raw products obtained was measured using a micro-hardness test device (Matsuzawa Co., Akita-ken, Japan). 

The 1D scanning mode applied in this experiment is illustrated in Figure 1. The hotspot created by the LMH applicator was continuously dragged along the printing axis and locally melted the powder. As the applicator moved on, the powder behind it was cooled down and solidified. This setup also enabled a stepwise scanning mode, in which the applicator made a sequence of consequent hotspots [46] (similar to spot welding) along the printing axis in the powder bed, forming a solid layer. The successive operation proceeded, layer by layer, in the vertical axis of the printed metal objects. This setup can also be operated in a *powder-on-demand* mode, in which the powder is specifically delivered during the process, only to the designated solid regions of the solid object [30,31,51]. 

## 3. Experimental Results

**Figure 3:**
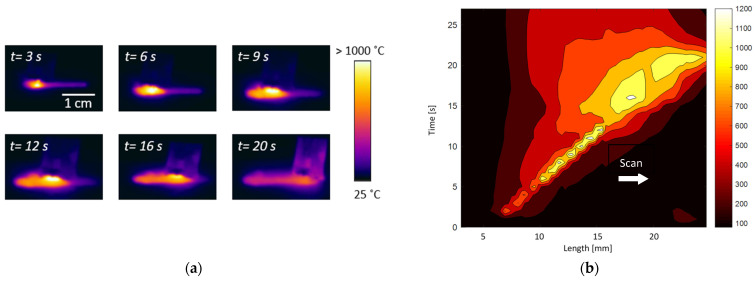
Temperature measurements of a scanning LMH–AM process along the 1D powder bed: (**a**) Thermal camera images in different times, showing the hotspot progress along the *x* axis. (**b**) The spatiotemporal evolution of the temperature along the scanned powder axis, measured by the thermal camera during the LMH–AM scanning process along the *x* axis vs. time. The scanning direction is indicated by the white arrow. The motion of the hotspot is evidenced by the yellow front line (>900 °C), propagating along the *x* and *t* dimensions at a ~1 mm/s speed.

**Figure 4:**
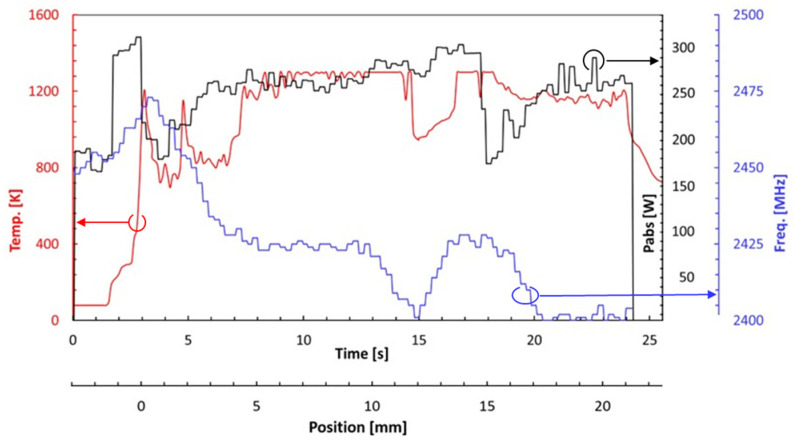
Measurements during a typical LMH scanning process, including the absorbed microwave power (in black), the frequency variation for the adaptive impedance matching (in blue), and the temperature that evolved along the scanned powder surface (in red; note that the thermal runaway instability is detected after ~3 s of microwave irradiation).

**Figure 5:**
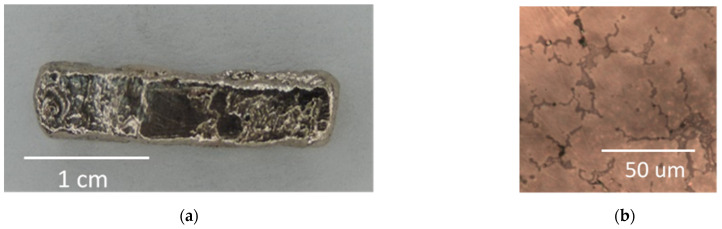
A ~25 mm long metal rod produced from powder (DirectMetal 20 [50]) by the scanned LMH–AM (**a**) and its porous structure (**b**).

Typical results of the continuous LMH–AM solidification process, investigated in these experiments, are presented in Figure 3, Figure 4 and Figure 5. A sequence of chronologic thermal images in Figure 3a shows the temperature evolution and the continuous hotspot motion along a ~20-mm solidified line in the powder layer. The spatiotemporal evolution of the temperature, measured by the thermal camera during the LMH–AM scanning process, is displayed by a contour plot in Figure 3b. The heat-affected zone (HAZ) follows the LMH-applicator scan, whereas the region beneath it exhibits a thermal runaway response as a moving hotspot, reaching the melting temperature while the previously scanned region behind it cools down and solidifies within a few seconds. The peak measured temperature (moving along the scanned path) is presented in Figure 4, with respect to the microwave-power absorbed by the metal powder and its frequency variations (due to the adaptive optimization applied for the impedance-matching tuning). A constant scanning speed of ~1 mm/s was applied in these experiments, at a 0.25-kW delivered microwave power. Some intermittent responses of the absorbed power (and consequent temperature drops) could be seen in between the longer, more stable periods. Figure 5a shows a typical product of the continuous LMH–AM experiment, namely a ~22×4×2 mm^3^ solid strip obtained from the metal powder in a single layer by the LMH–AM scan. Its porous structure is presented in Figure 5b, as observed by an optical microscope.

**Figure 6:**
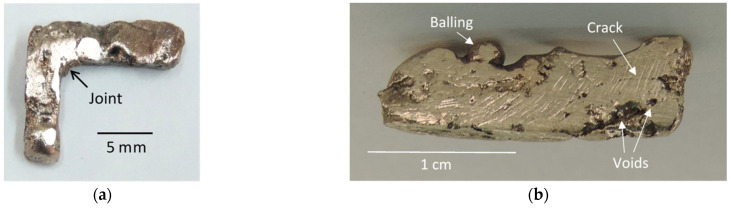
Experimental examples of scanning LMH–AM products: (**a**) An L-shape metal rod fabricated by two straight rods, each produced by scanning LMH–AM and joined together by a powder-batch LMH. (**b**) A metal object (~20×4×6 mm^3^) made of 5 powder layers by scanning LMH–AM in five subsequent scans. This result demonstrates both the solidification capability of the scanning LMH–AM and the imperfections encountered in this process. These defects, including balling, cracking, and voids, are partly attributed to the fixed (non-adaptive) scanning velocity.

An L-shaped rod obtained by joining two sections produced by continuous LMH–AM scans is shown in Figure 6a. The joining was achieved by an additional powder batch (~8 mm^3^ in volume) placed in the corner between the two sections and subjected to LMH as in [32,33]. The continuous LMH–AM scan was applied in the vertical dimension (*z* axis) in a layer-by-layer solidification, where each powder layer added a ~1–2 mm thickness to the solidified object. Figure 6b shows a ~20×4×6 mm^3^ metal bar produced by five layers in successive LMH–AM vertical cycles. The feasibility of layered LMH–AM scanning is demonstrated by the rigid object obtained, but it also reveals some of the imperfections that might be encountered at the present experimental stage of the scanning LMH–AM studies. However, these imperfections include local defects of balling, voids, and cracks (as marked in Figure 6b).

**Figure 7:**
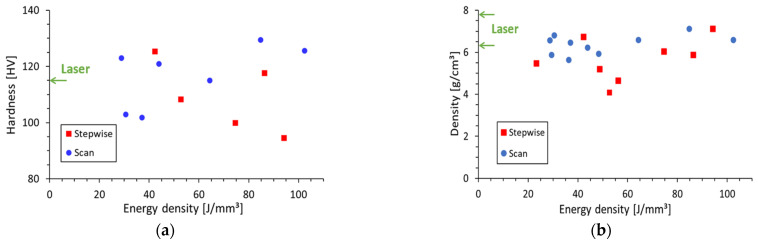
(**a**) Density and (**b**) hardness test results of scanning and stepwise LMH–AM products (as in Figure 5) vs. the spent microwave energy per unit powder volume (i.e., the energy density). The green arrows indicate typical laser-based AM specifications [50], as a benchmark.

The mechanical properties of the manufactured samples were examined in order to evaluate the properties of the fabricated materials. The product density measurements, performed by the Archimedes revisited technique [52], resulted in ~6.5 g/cm^3^ on average, as shown in Figure 7a. This LMH–AM product density is comparable to the ~6.3 and 7.6 g/cm^3^ densities specified for laser-based AM products in their cores and surfaces, respectively, by the powder manufacturer [50]. The measurements also show a slight advantage in this aspect of scanning LMH–AM products over those obtained by stepwise LMH [32,33].

Local hardness measurements of scanning LMH–AM solidified strips were performed by micro-hardness tests, using a Vickers indenter with a 0.3-kg load for 15 s, at a cross-section cut along the strip product. The hardness results are shown in Figure 7b for various power levels and scanning speeds (represented in a unified manner by the spent microwave energy per powder volume, namely the energy density) obtained in LMH scan and stepwise operations. The LMH–AM hardness results, ranging from 90 HV to 130 HV, are comparable to the 115-HV hardness specified for the laser sintering of this powder [50].

**Figure 8:**
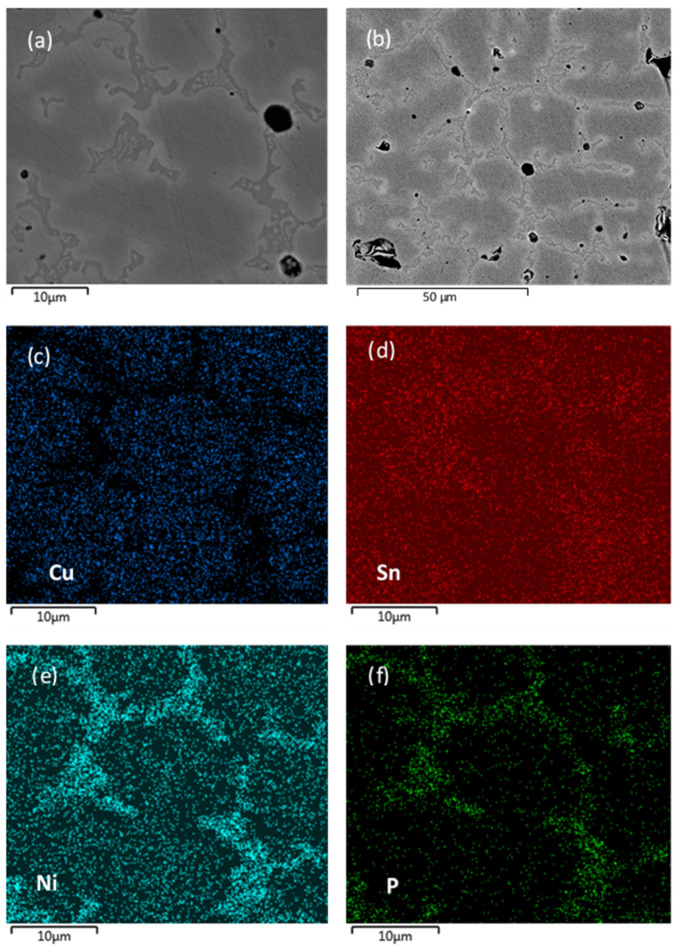
SEM and EDS images, showing a typical scanning LMH–AM grain structure (**a**,**b**), and the non-uniform spatial distribution of the main powder ingredients copper, tin, nickel, and phosphorous, in (**c**–**f**), respectively. A mutually complementing pattern, typical to selective melting processes, is observed in this case.

The LMH–AM products, in the form of solidified strips and layered structures, were examined by SEM. Typical SEM observations of a scanning LMH–AM product are presented in Figure 8a–f. The micro-structure is characterized by a typical grain size of 21±9 μm, as measured on 12 grains in Figure 8b. Most of the obtained pores of a rounded shape are distributed at the interface between the grains, i.e., at the segregation region (Figure 8a,b). The rounded shape of the pores is due to the gas entrapment during sintering, while the irregularly shaped cavities could be referred to as pore aggregation. The pore regions have a linkage to weak mechanical properties. The relative occupation of the pore region is deduced as 3% by the image processing of a planar surface sample of a 10^5^ µm^2^ area. The EDS mapping in Figure 8c–f reveals the spatial distributions of the sample’s main elements, namely copper (Cu), tin (Sn), nickel (Ni), and phosphorous (P). Copper mainly accumulates in the dendrites and the grains, as seen in Figure 8c, whereas the tin, nickel, and phosphorous ingredients are mostly in the matrix between the copper grains and in the inter-dendrite phase (Figure 8d–f). The EDS normalized atomic element analysis for the grains provides Cu (89%) with additives of Sn (1.5%) and Ni (9.5%), while EDS between the grains provides 19.7%, 7%, 55.4%, and 17.9% for Cu, Sn, Ni, and P, respectively. In addition to the bronze phase at the grains, the segregation area is composed of high Ni and P concentrations with the additive of Sn. The high Cu content at the grain site could affect the measured Cu content at the boundaries, due to sub-micron EDS lateral resolution (at 20 kV electron beam), and thus, the phase identification at the grain boundaries would require further analysis. These non-uniform, mutually-complementing distributions are attributed to the higher melting temperature of copper, which is not fully melted, whereas the tin and phosphorous (with lower melting temperatures) and nickel fill in the gaps among the grains. Similar effects were observed in previous (stepwise) LMH–AM experiments using a ∼1-kW magnetron [30,32] as well as a ~0.25-kW solid-state generator [33]. These observations also resemble the phenomena of selective alloying, segregation, and precipitation, known in SLM and EDM mechanisms [6].

**Figure 9:**
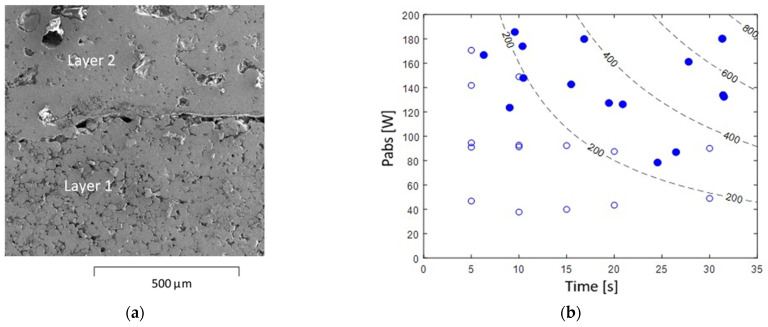
Layer bonding quality: (**a**) A SEM image of the layered structure, with an imperfect bonding between two layers. (**b**) The absorbed microwave power vs. the total LMH–AM process duration required for a proper bonding of the melted powder layer to the underlying previously solidified layers (the full and empty dots indicate proper and improper bonding results, respectively). The energy density (in J/mm^3^) is marked by the dashed curves.

A typical SEM image of the interface between adjacent layers is shown in Figure 9a for a partially imperfect bonding between two layers (as marked, for instance, in Figure 6b). The adjacent layers were characterized on the macroscopic level, i.e., over a 1-mm length scale, as well as on the micron scale, showing cavities along the interface between the layers. The LMH conditions required for a sufficient quality of both solidification and bonding were investigated in a static LMH mode. The results obtained by 28 runs in various conditions of absorbed power and exposure time are shown in Figure 9b (the solid and hollow dots indicate successful and failed joining attempts, respectively). The constant energy–density contours in the graph indicate that the bonding quality is insufficient below ~200 J/mm^3^. An energy density level of ~300 J/mm^3^ is sufficient for a reasonable bonding, preferably by exposures to relatively higher power levels for shorter periods. However, the optimal irradiation in this case, of a ~8 mm^3^ powder’s volume exposed, is estimated as ~150 W for a ~20 s exposure period. A higher power level (above ~200 W in this case) may cause structural deformations and even plasma excitation. It is noted, however, that a proper layer bonding to a solid substrate requires a higher energy density than that needed for a single-layer solidification (~300 vs. ~80 J/mm^3^, respectively).

The scanning LMH–AM applicator was tested in similar conditions, also with a uniform powder bed, without the two side supports, for the powder shown in Figure 1. The results obtained were found to be similar to those shown in Figure 5, except that the metal strips were wider than those produced with the side supports (~6 mm as compared to ~2–4 mm, respectively). The width of the metal strips obtained without side supports is actually dictated by the applicator’s diameter (~6 mm in this case), as was also verified with a wider applicator. 

## 4. Theoretical Analyses

The continuous LMH scanning process illustrated in Figure 1 was simulated by a coupled thermal-electromagnetic (EM) model [53]. The powder’s dielectric and magnetic properties were simulated in [33] using the *effective-medium* method [15,54]. The microwave absorption of the metallic powder was dominated by a magnetic loss mechanism, attributed to eddy currents on the outskirts of each particle [30,31,32,33]. Due to the finite electrical resistivity (further increased with temperature), the currents penetrated into the particles and broadened their EM skin depth [15]. The main cause for the microwave-power dissipation in this metallic powder is therefore represented by its effective (macroscopic) complex magnetic permeability, $\mu \left(T\right)=\mu^{\prime }-j\mu^{\prime \prime }$.

The metallic powder was simulated as a lattice of ~20-µm diameter particles with small isolating gaps between them [54]. The particles were arranged in a face-centered cubic (FCC) structure, terminated by two microwave ports. The microwave-scattering parameters (*S*_11_ and *S*_21_ at these ports) were simulated in order to extract the macroscopic EM properties of the metallic powder [30]. These included the temperature-dependent complex magnetic permeability and the effective dielectric permittivity, $\varepsilon_{e}\left(T\right)={\varepsilon^{\prime }}_{e}-j{\varepsilon^{\prime \prime }}_{e}=\varepsilon^{\prime }-j\left(\varepsilon^{\prime \prime }+\sigma_{e}/\omega \right)$, where ω is the operating angular frequency, and $\sigma_{e}\left(T\right)$ is its electric conductivity [55]. In this procedure, the powder’s magnetic permeability, for instance, was approximated at $T<T_{m}$ by the second-order polynomial expression

(1) $$\mu \left(T\right)=\mu_{0}\left[0.3384+3\times 10^{-4}T-9\times 10^{-8}T^{2}+j\left(0.0565+2\times 10^{-4}T-7\times 10^{-8}T^{2}\right)\right]$$

where $\mu_{0}=4\pi \times 10^{-7}\;H/m$ is the vacuum magnetic permeability. In this analysis, the electric conductivity became more significant near the melting temperatures. The simulated powder’s parameters were substituted into the macroscopic LMH model [30,53], which couples the EM wave equation, the heat equation, and the dissipated power density, as follows:

(2) $$\nabla \times \nabla \times \tilde{H}-\omega^{2}\mu \varepsilon_{e}\tilde{H}=0$$

(3) $$\rho c_{p}\frac{\partial T}{\partial t}-\nabla \cdot \left(k_{th}\nabla T\right)=Q$$

(4) $$Q=\frac{1}{2}\omega \left[\mu^{\prime \prime }{\left|\tilde{H}\right|}^{2}+{\varepsilon^{\prime \prime }}_{}^{}{}_{\mathrm{eff}}{\left|\tilde{E}\right|}^{2}\right]$$

where $\tilde{H}$ and $\tilde{E}={\left(j\omega \varepsilon_{e}\right)}^{-1}\nabla \times \tilde{H}$ are the magnetic- and electric-field vector components of the phasor EM wave, respectively. In the heat Equation (3), the effective powder density, ρ, the heat capacity, $c_{p}$, and the thermal conductivity, $k_{th}$, characterize the temperature-dependent properties of the metal powder. The microwave power-density absorbed in the powder, *Q* (4), couples with the EM wave and the heat equations (Equations (2) and (3), respectively).

The temperature profile *T* was spatiotemporally evolved at a much slower rate than that of the EM wave variations (note that the thermal response time, >1 ms, was approximately six orders of magnitude slower than that for the EM period). Therefore, the two-time-scale approximation was applied here, which allowed for the mixed presentation of the rapid EM wave variations in the frequency domain [53] and the relatively slow-varying temperature evolution in the time domain, both in the same set of equations.

**Figure 10:**
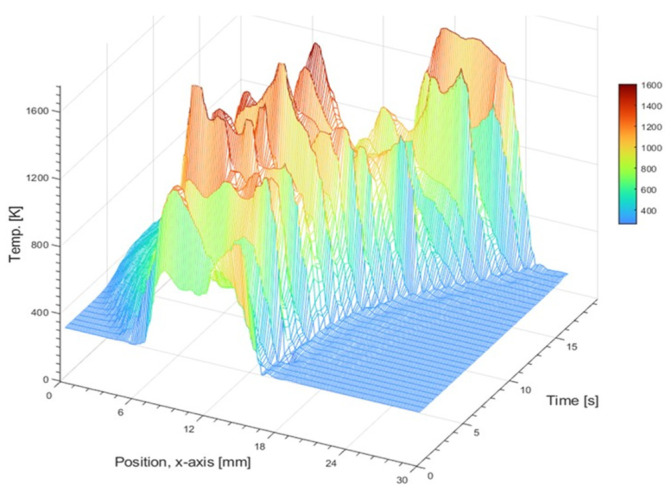
A theoretical simulation of the spatiotemporal evolution of the temperature profile during a scanning LMH–AM process (applied to a ~20×2×1 mm^3^ powder layer, by a 200-W LMH applicator, at a ~1 mm/s scanning speed).

The coupled EM–thermal model (1–4) was used to simulate the evolution of the hotspot temperature profile due to the LMH effect within the powder bed, as well as the spatiotemporal evolution due to the applicator’s scanning motion (in a moving mesh mode). Figure 10 shows the simulated temperature profile during a scan of a ~20×2×1 mm^3^ powder strip (in a 1D powder bed) with a 200-W microwave power, at a ~1 mm/s scanning speed. The numerical results of this example show that the HAZ, moving along with the applicator, reaches the melting temperature beneath the electrode, while the scanned region behind it is cooled down (similarly to Figure 3b). The spatiotemporal evolution of the temperature profile along the scanned path supports, in this aspect, the feasibility of metal-powder solidification by a continuous scanning LMH process, at the relatively low power level (~200 W) available with solid-state applicators. 

## 5. Discussion

This study demonstrates the feasibility of metal-powder solidification by LMH in a continuous scanning mode, layer by layer, which to some extent mimics the common laser-based AM techniques. The experiments presented here are, however, limited to a 1D scan (along the *x*-axis in Figure 1), whereas the powder is confined by mechanical supports in the other lateral (*y*-axis) dimension. The experimental results show the fundamental capability of LMH–AM scanning in the production of basic elements, such as metal rods in line- and L-shapes.

The mechanical properties of the scanning LMH–AM products show comparable density and hardness to laser-based AM with the same powder (∼7 g/cm^3^ and ~115 HV, respectively). The dissipated LMH energy densities required for the powder solidification and layer bonding are ~0.1 kJ/mm^3^ and ~0.2 kJ/mm^3^, respectively (similar to those of laser-based AM), which result in a yield efficacy in the order of ~10 Wh/g. In view of the higher overall efficiency of microwave generators, and their lower cost (per watt) compared to lasers, the LMH–AM could be preferable in this regard. On the other hand, its relatively low resolution and imperfect surface quality are significantly inferior with respect to laser-based AM techniques (due to the relatively long microwave wavelength, ~10^−1^ m vs. ~10^−6^ m in lasers, which implies a larger HAZ). However, preliminary studies show that LMH at 2.45 GHz can be focused into a ~1-mm diameter HAZ and even smaller sizes. Furthermore, future LMH–AM applicators may utilize higher microwave and millimeter wave frequencies to form narrower hotspots and higher resolutions. One may also hypothesize, for future study, that the contactless magnetic confinement, demonstrated before for ferroelectric-metal powder in batch LMH mode [32], may also improve the resolution in a scanning LMH–AM mode.

The relatively low power required for the LMH–AM applicator (~0.2 kW) can be generated by advanced solid-state amplifiers. These devices open new possibilities for adaptive impedance matching and controls, which may further improve the product quality of the scanning LMH–AM technique. For instance, the frequency tunability of the solid-state generator (unlike the fixed-frequency magnetron) enables the instantaneous impedance-matching adjustment applied here in a feedback loop. Similarly, the intermittency observed in the experimental and theoretical LMH–AM scanning results here (e.g., in Figure 4, Figure 7 and Figure 10, respectively, attributed to the thermal runaway instability) can be stabilized by additional feedback loops (e.g., by adaptive controls on the instantaneous scanning speed and input microwave-power). This adaptive feature may also alleviate the imperfections observed (e.g., in Figure 7).

## 6. Conclusions

The LMH–AM studies, in both stepwise (batch) and continuous modes (in [30,31,32,33] and here, respectively) do not seem to introduce any competition to laser-based AM techniques in the construction of delicate structures. However, LMH–AM may present economic advantages in the construction of relatively large and rough metal structures, e.g., in Big Area Additive Manufacturing (BAAM) applications [56] (note that solid-state LMH–AM devices can be integrated in multi-applicator arrays to increase their throughput). In more delicate operations, the solid-state LMH–AM could be integrated with laser-based AM in order to improve the overall process efficacy of the system. In such hybrid schemes, the LMH could be effectively used to build the underlying rough construction of the main body, whereas the laser could be more effectively used to more accurately complete the fine details. 

Another hybrid approach may stem from the recently found synergic interaction of direct current (DC) and LMH in metals [38]. This mutually-enabled DC–LMH effect leads to a hotspot formation and localized melting deeper than the surficial skin-depth limit in metal bulks. In metal powders, the combined DC–LMH process may enhance synergic flash-sintering effects [57,58,59] and hence improve the controllability and performance of microwave-based AM processes. 

Scanning LMH may introduce advantages also for sintering in molds, as compared to volumetric microwave heating, in terms of better uniformity of the product, in a combined approach of *AM-in-mold*. For AM without molds, LMH can also be applied in *powder-on-demand* or *wire-on-demand* modes, as proposed in [31] and demonstrated in [60], for reinforced plastics. These modes may save the layer-by-layer powder-bed complexities, e.g., in handling, costs, and safety of handling metal powders in large quantities.

The present study adds the scanning mode to the LMH–AM paradigm [30,31,32,33]. Additional studies are still required, however, in each of the R&D paths proposed above, in order to explore novel LMH–AM schemes and improve their performance to the level required in practice. However, this study demonstrates the potential feasibility of this low-cost LMH technique as a compact and effective means for the AM of metal parts.

## Data Availability

The data presented in this study are available on request from the corresponding author.
